# Editorial: The path towards achieving gender equity for surgeons: The role of individuals, their professional organizations along with the associated healthcare systems

**DOI:** 10.3389/fsurg.2022.1056655

**Published:** 2022-11-25

**Authors:** Deborah J. Verran, Maria Irene Bellini, Katrin Rabiei

**Affiliations:** ^1^ Ramsay Healthcare, Sydney, NSW, Australia; ^2^ Department of Surgical Sciences, Sapienza University, Rome, Italy; ^3^ Institute of Neuroscience and Physiology, University of Gothenburg, Gothenburg, Sweden

**Keywords:** gender, surgeons, equity, health systems, surgery

**Editorial on the Research Topic** The path towards achieving gender equity for surgeons: The role of individuals, their professional organizations along with the associated healthcare systems

When the editorial support team at Frontiers in Surgery were looking for a research topic with a focus on gender equity within and across the various surgical disciplines, some of us answered the initial call. This was followed by us working out which of the 14 sections of the journal would be the best fit for this particular research topic, with Visceral Surgery subsequently being decided upon. A collective decision was also made early on in the process to deliberately target a full range of all of the relevant sub-topics for potential manuscripts with the aim of facilitating the publication of as much in the way of relevant research along with perspectives from around the world. Plus, it was anticipated at an early stage that there would be additional challenges related to the COVID-19 pandemic which may also be a focus for some authors (1).

What led to this decision being made by the team at Frontiers in Surgery? It had not gone unnoticed that over the last 5 years there had been a steady increase in the number of published manuscripts with a focus on gender equity appearing not only in high-profile medical journals (2) but also in surgical journals (3). This is of relevance not only to individual surgeons but also to the institutions within which they practice along with the relevant professional surgical societies (4, 5). In addition, it is now understood that having a surgical workforce made up of individuals who reflect the demographics of the community which it serves (6) is also important in ensuring that health equity can be achieved (7).

The secondary impacts of the ongoing COVID-19 pandemic on healthcare systems created challenges for everyone involved in this research topic whether it be the editors, the reviewers, or the authors. We received feedback on multiple occasions attesting to this. Nevertheless, of the close to 30 groups of authors who initially showed an interest in the research topic, 10 manuscripts were eventually able to be published.

Medical and surgical education emerged as a sub-topic of significant ongoing interest, with 5 manuscripts being published. Some of the factors which contribute to medical students having negative perceptions when it comes to considering a career in surgery are examined both for Orthopedics (Hull et al.) and Urology (Reale et al.). Then there is the data on the relatively high prevalence and impact of microaggressions on medical and surgical trainees in the Gulf regions (Al Rashed et al.), regardless of gender. The challenges being wrought by the COVID-19 pandemic were the focus of one manuscript (Cimen et al.), on how the continuing professional development of surgeons needed to be shifted to an online/virtual format. The small numbers in this particular study meant that no differences were able to be detected according to gender for the outcomes of interest. Surgical education was also a focus of one manuscript from Mexico (Mejia Fernandez et al.), on the rates of female participation in the annual scientific meeting of their national General Surgical association. They detect some differences, some of which are related to the proportion of women who are undertaking surgical training. The authors make a number of interesting recommendations that will be of particular interest to surgeons involved in the running of scientific meetings.

The working environment was the predominant focus of the remaining 5 manuscripts. The subgroup analysis of the surgeons who responded to a survey on doctors' experiences including with infertility and pregnancy in Australia (Kevric et al.) was both interesting and revealing. Anyone with an interest in family leave entitlements should look up this manuscript. Fewer female surgeons being involved in academic research, particularly in clinical trials formed the basis of another manuscript from Australia (Thao Luong et al.) along with one from the United States (Venkatesh et al.). The implications of this were explored in detail in each case, with the data from the United States also revealing a lack of diversity of the researchers in the sub-group of trials focusing on traumatic brain injury. This also appeared to be associated with the demographics of the patients being recruited into these particular trials, with the ensuing implications for achieving health equity. Finally, there was a focus on the extent of either bullying and/or harassment being encountered by female surgeons in the workplace whether it be in Gynecological Oncology in the United States (Hong et al.), or in Surgical Oncology in India (Pandrowala S). In each case, the authors make a series of system-level recommendations on how to both address and deal with these undesirable behaviours.

As can be seen, many of these manuscripts are potentially of interest to medical educators, researchers, clinicians who hold leadership positions within organizations, and of course all of the surgeons who are involved in any of the aforementioned activities. The most important common theme which is apparent from all of this is the real need to both develop and implement systemic type measures to deal with the issues, depending on where they lie, whether it be within an organization and/or professional societies. Finally, we would like to thank the authors who kindly submitted their work, the reviewers who so graciously gave their time, and the team in the Frontiers in Surgery support office. We hope that this research topic stimulates ongoing interest such that other related manuscripts continue to be submitted to Frontiers in Surgery.
